# Retinoic acid regulates the proliferation, differentiation, and cell death of limb skeletal progenitors, contributing to establish the size and identity of the digits

**DOI:** 10.1186/s13227-025-00248-4

**Published:** 2025-07-09

**Authors:** Cristina Duarte-Olivenza, Goretti Moran, Juan M. Hurle, Juan A. Montero, Carlos I. Lorda-Diez

**Affiliations:** 1 Departamento de Anatomía y Biología Celular, Facultad de Medicina, Universidad de Cantabria, C/Cardenal Herrera Oria s/n, 39011 Santander, Spain; 2 IDIVAL, 39011 Santander, Spain

**Keywords:** Morphogenetic cell death, Citral, Retinoic acid gradient, Raldh2, Cyp26a1, Digit identity, Digit outgrowth

## Abstract

**Background:**

The development of the digits (fingers/toes) provides an excellent model for analyzing the molecular regulation of skeletal morphogenesis in vertebrates. Digits develop in the autopod as radial chondrogenic condensations separated by interdigital spaces containing undifferentiated skeletal progenitors destined to die by apoptosis. In avian species, leg digits are characterized by a differential size, with the first digit being short and the fourth largest.

**Results:**

In vitro experiments using micromass cultures of digit progenitors demonstrated that RA controls the balance between cell death, cell proliferation, and cell differentiation in a dose-dependent fashion. In vivo, qPCR analysis revealed that the RA-synthesizing enzyme *Raldh2* and the RA-degrading enzyme *Cyp26a1* are expressed in the interdigits in an inverse gradient that correlates with the size of the digit adjacent to each interdigit. RA gain- and loss-of-function experiments via pharmacological approaches confirmed a close correlation between interdigital RA and digit size. A low concentration of RA applied to the first interdigits, when the phalanxes of the first digit are being formed, promoted mesodermal cell proliferation and caused elongation of digit 1, while blocking RA synthesis into the third interdigit inhibited cell proliferation, followed by a reduction in the size of digits 3 and 4.

**Conclusions:**

This study reveals a potential role for Retinoic Acid (RA) expressed in the interdigits in the regulation of the differential digit size. The morphological similarity of the digit patterns obtained in our experimental assays with those of other tetrapods suggests an evolutionary role of RA in determining digit morphology.

**Supplementary Information:**

The online version contains supplementary material available at 10.1186/s13227-025-00248-4.

## Introduction

Retinoic Acid (RA) is a major regulator of vertebrate skeletal morphogenesis that functions via a complex signaling cascade that modifies the expression of target genes [15, 68]. Active RA metabolites are produced in the embryonic tissues from vitamin A (all-trans-retinol) via retinol alcohol dehydrogenases (ADHs: 1, 3 and 4) and retinol dehydrogenases (RDHs: 1 and 10), which form retinal. Retinal is next oxidized, in a spatiotemporally regulated fashion, by retinaldehyde dehydrogenases (Raldh: 1, 2, and 3), forming Retinoic Acid (RA) [15]. RA, is stored in the cells by retinoid-binding proteins that transport it from the cytosol to the nucleus [54]. In the nucleus, RA binds to nuclear retinoic acid receptors RARα, β, and γ or to the orphan nuclear receptor PPAR ß/∂ (peroxisome proliferator-activated receptor beta delta), which, upon binding to RA, forms heterodimers with retinoid X receptors (RXRα, β, γ) that regulate the transcription of target genes bearing RA response elements (RAREs) [42, 46, 54]. The function of RA is also modulated in a spatiotemporal fashion by CYP26 enzymes that metabolize and inactivate retinoic acid [49], determining the exposure of target cells to RA.

Although still controversial [76], evidence indicates that RA in the embryonic trunk and limb tissues is required for the formation and growth control of limb primordia and for proximodistal patterning of the limb skeleton [5, 7, 29, 36, 40–42, 52, 60, 72]. In addition to its signaling function, RA is involved in cell differentiation [43, 69] and tissue remodeling associated with limb morphogenesis [12, 50, 73, 75]. Thus, RA participates in the differentiation of limb tissue components, including cartilage [11, 13, 32, 63], musculo-tendinous progenitors [13, 43, 50], and joints [70], and, in the regression of interdigital tissue [2, 17, 31, 51, 75].

The remodeling of the interdigital tissue of tetrapod embryos is a most illustrative example of a sculpting role of cell death in morphogenesis. The process has been analyzed via genetic and classical embryological approaches. These studies identified FGF [44], BMP [4, 30, 78, 79], WNT [22], and RA signaling [8, 12, 17, 21, 24, 31, 51, 73, 75] as major directors of the degenerative process.

The role of RA signaling in limb developmental cell death is supported by genetic and pharmacological approaches [8, 12, 17, 21, 24, 31, 34, 51, 72, 75]. The mechanisms proposed to explain the pro-degenerative role of RA in the developing limb are as follows: (i) dysregulation of the expression of transcription factors responsible for positional information of skeletal progenitors [25]; (ii) direct activation of the apoptotic molecular machinery [24]; (iii) upregulation of proapoptotic growth factors of the BMP family, including BMP7 [17, 75]; and (iv) antagonism of the survival influence of FGF signaling [24].

The present study was conducted to gain insights into the biological influence of RA on interdigital tissue remodeling and digit morphogenesis via a combination of in vitro and in vivo approaches. The balance between cell death, cell proliferation, and cell differentiation was analyzed in micromass cultures as an organoid-like assay [14, 18]. The in vivo function of retinoic acid in digit morphogenesis was evaluated by interdigital targeted gain- and loss-of-function experiments via pharmacological approaches.

## Materials and methods

Rhode Island chicken embryos ranging from 4 to 9 days of incubation, stages 24 to 36 of Hamburger–Hamilton [23] were used. The embryo extraction was performed following the ethical recommendations of the European Communities Council.

### In vivo experiments

#### Embryo treatments

To analyze the influence of RA in the development of the digits we performed gain-of-function experiments via local treatments with all-trans-retinoic acid (atRA), and loss-of-function experiments with Citral (C_10_H_16_O, Fluka) to inhibit Raldh2 [63]. Eggs were windowed at the desired stages and treatments were performed via interdigital implantation of micro-beads bearing different concentration of the drugs diluted in DMSO. atRA was administered with AG1X-2 (BioRad) beads incubated for 1 h in 25 to 200 μg/ml atRA (Sigma). Citral was administered with SM2 beads (BioRad) incubated for 1 h in 20 mg/ml Citral (Fluka). All the experiments were contrasted by parallel experiments implanting DMSO soaked beads from which no effects were observed. Bead implantation was performed between stages 24–28HH., and the embryos were further incubated and processed at the appropriate stages for morphological study, and analysis of cell proliferation and a apoptosis. The location of the beads at the desired regions was confirmed in 1 or 2, embryos of each experiment few hours after surgery by direct microscopic exam of fixed samples, or after in-situ hybridization with *Sox9* that is a precocious marker of the digit rays.

#### Morphology

The skeletal morphology of the experimental limbs was studied in whole-mount specimens after cartilage staining with Alcian Blue (AB).

#### BrdU incorporation and TUNEL assay

Cell proliferation was analyzed by bromodeoxyuridine immuno-labeling. For this purpose, 100 µl of bromodeoxyuridine (BrdU) solution (100 mg/ml) was injected into the amniotic sac. After 30 min of further incubation, the embryos were sacrificed. The autopod was then dissected free, fixed in 4% paraformaldehyde sectioned at 100 µm with a vibratome, and processed for immune-labeling. Monoclonal mouse antibody against bromodeoxyuridine was employed (Amersham Biosciencies). Samples were examined with a laser confocal microscope (LEICA LSM 510).

Apoptosis was evaluated by TUNEL assay, using the “in-situ” cell death detection kit (Roche) in vibratome sections processed as above.

#### In-situ hybridization studies

In-situ hybridizations for *Sox9*, *Raldh2*, *RARα*, *RARβ*, and *RARγ*, *PPAR ß/∂*, and *Cyp26a1* were performed in whole-mount specimens or in 100 µm vibratome sections. Samples were fixed in 4% PFA and treated with 10 mg/ml of proteinase K for 20–30 min at 20 °C. Hybridization with digoxigenin labeled antisense RNA probes was performed at 68 °C. Alkaline phosphatase-conjugated anti-digoxigenin antibody dilution 1:2000 was used (Roche). Reactions were developed with BCIP/NBT substrate as the chromogene (Roche). Specific primers are provided on request.

### In vitro studies

#### Micromass culture and tissue processing

The undifferentiated tissue of the distal margin (*progress zone*) of the leg buds of stage 25 embryos (4.5 incubation day; id) limb, was dissected free and cells dissociated by digestion with 0.25% trypsin (Sigma) and 0.25% collagenase (Worthington). After filtering through a 70-μm strainer (Miltenyi Biotec) to remove ectodermal debris and clumps of undissociated tissue, the cells were re-suspended in Dulbecco’s modified Eagle medium (DMEM, Lonza) at a concentration of 3 × 10^5^ cells/ml. Ten microliter drops were pipetted into each well of a 48‐well plate (Thermo Scientific) and allowed to attach for 2 h, and then 200 μl of DMEM was added to each well.

#### Retinoic acid and Q-VD-OPH treatments

Micromass cultures were treated by adding to the medium all-trans-Retinoic acid (atRA) diluted in ethanol 100% to reach concentrations ranging from 5 to 500 ng/ml. Treatment was maintained for 36 or 48 h.

To check the apoptotic nature of cell death in the cultures, micromasses were treated during the last 12 h of culture with 20 µM Q-VD-OPH diluted in DMSO to inhibit caspases [6] and processed for flow cytometry.

In all cases the dilution solution was added at the same concentration to the control cultures.

#### Flow cytometry

Samples from control and treated micromass cultures were dissociated using trypsin–EDTA (Lonza). Tubes containing approximately 1 × 10^6^ cells were fixed in 90% ethanol and subsequently incubated overnight at 4 °C in a propidium iodide (PI) staining solution composed of 0.1% sodium citrate, 0.01% Triton X-100, and 0.1 mg/ml propidium iodide (Sigma). DNA content analysis for cell cycle and cell death assessment was performed by flow cytometry using a FacsCanto cytometer (Becton Dickinson), and data acquisition and analysis were carried out using CellQuest software.

### Real-time quantitative PCR (qPCR) for gene expression analysis

Differences in the expression of the RA-synthesizing enzyme *Raldh2*, and the RA-degrading enzyme *Cyp26a1* and the receptors *RARα*, *RARβ*, and *RARγ*, and *PPAR ß/∂*, during the stages of digit formation were analyzed by SYBR Green qPCR in interdigital tissue samples isolated microsurgically, and also in samples of the distal margin mesoderm of stage 24 limbs. Each sample set consisted of 10 interdigits or tissue fragments. RNA was extracted using the NucleoSpin RNA kit (Macherey–Nagel). cDNA was synthesized by RT-PCR using random hexamers with the RevertAid RT Kit (Thermo Scientific). The cDNA concentration was measured in a spectrophotometer ND-1000 (Nanodrop Technologies) to be adjusted to 0.5 μg/μl. SYBR Green-based qPCR experiments were performed employing SYBR Select Master Mix (Life technologies) using the CFX Connect Real-Time System (BioRad) with automation attachment. Specificity was checked by the presence of single peaks in the dissociation curves. Mean values for gene expression fold changes were calculated relative to a calibrator. qPCR chicken specific primers are included in Supplementary Table S1. *Rpl13* had no significant variation in expression across the sample sets, and, therefore, was chosen as the normalizer housekeeping gene in these experiments.

### Statistics and reproducibility

All the measurements included at least three independent experiments performed under the same conditions. Statistical analyses were performed using GraphPad Prism 5 (GraphPad). The results are expressed as means ± standard deviation. Paired analyses were performed using the *F* test followed by Student’s *t* test. The differences among three or more sample groups were evaluated through one-way analysis of variance ANOVA with Bonferroni post-hoc test. *p < 0.05, **p < 0.01, and ***p < 0.001 denoted statistical significance.

## Results

### RA promotes cell death and modulates the chondrogenesis of digit skeletal progenitors in vitro

High cell concentration cultures of limb skeletal progenitors termed "micromass cultures", is a widely accepted assay that replicates in vitro the events leading to limb skeletal differentiation, except for the influence of morphogenetic signals that confer topographic differences to the appendicular skeleton due to their inhomogeneous distribution within the limb bud. Consistent with previous studies, the addition of atRA to micromass cultures of limb skeletal progenitors modified the pattern of chondrogenic differentiation and increased the rate of cell death [11, 25, 48, 67, 74, 77]. Both effects were dose- and time dependent.

**Fig. 1:**
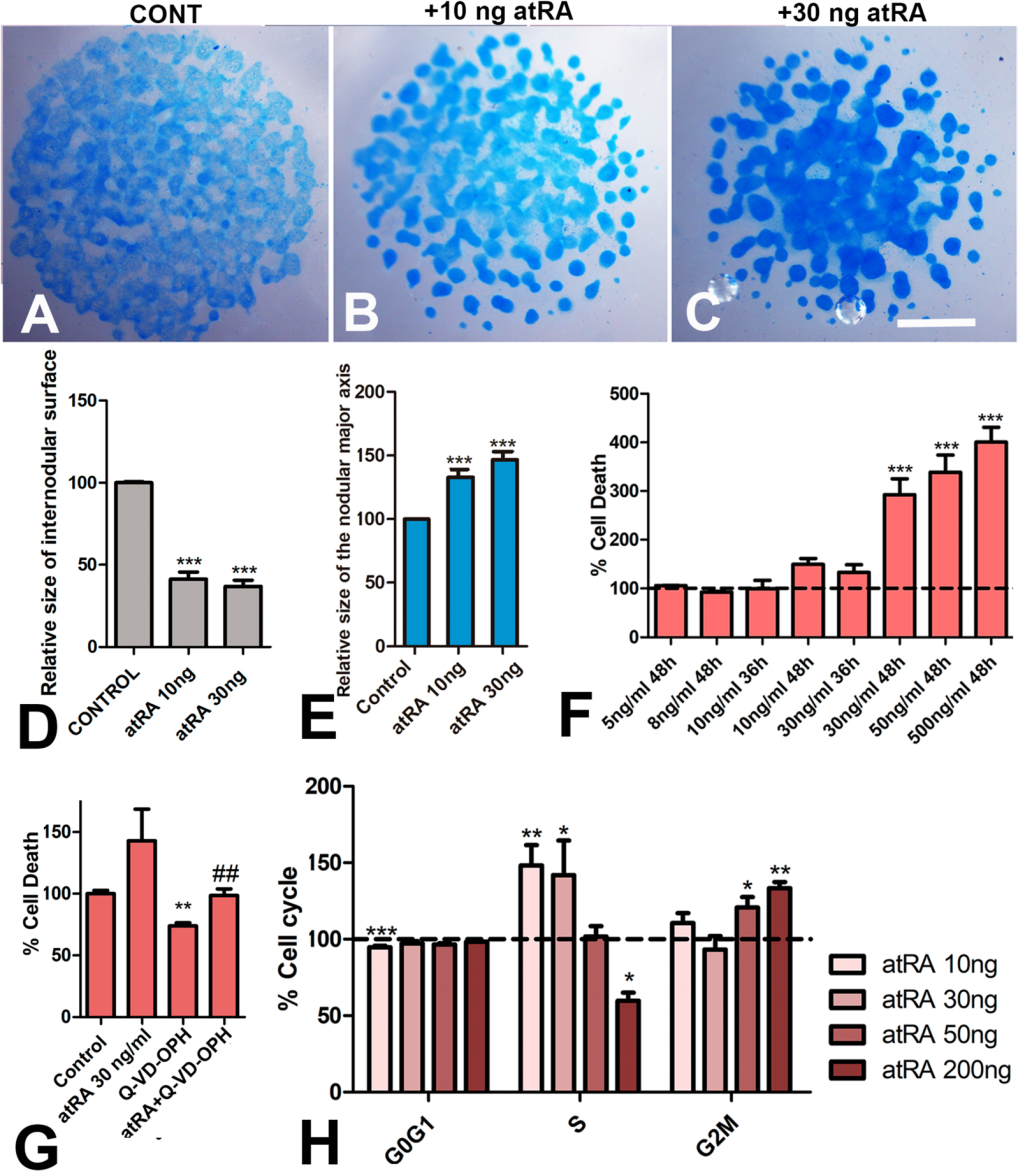
Effects of atRA treatments in micromass cultures. **A**–**C** Control (**A**), and atRA-treated (**B**, **C**) 6-day-old micromass cultures of autopodial skeletal progenitors stained with Alcian Blue. Treatments were performed for 48 h starting at time 0. (**A**), control; (**B**), micromass treated with 10 ng/ml atRA; **C** micromass treated with 30 ng/ml atRA. Note the increased staining intensity of the cartilage nodules and the increased internodular surface lacking Alcian Blue staining in the treated cultures. **D** Comparative size of the internodular surface in 6-day control and atRA-treated micromasses. **E** Comparative size of the major axis of the cartilage nodules in 6-day micromass cultures treated with 10 and 30 ng/ml of atRA. Data are based on measures of 100 nodules from at least 3 distinct cultures. **F** Intensity of cell death in 2-day cultures after atRA treatment at different doses and stages of culture. The dotted line represents values under control conditions. **G** Graphical representation of the relative number of dead cells (red) in 2-day control and atRA-treated cultures after the addition of 20 µM Q-VD-OPH for the last 12 h of culture. The experimental cultures were treated with 30 ng/ml atRA during the first 36 h of culture. **H** Graphic representation of cells at the different phases of the cell cycle in cultured micromasses treated for 48 h with atRA at 10, 30, 50 and 200 ng/ml. Scale bars in A, B, and C = 1 mm. Statistical significances: *p < 0.05; **p < 0.01; ***p < 0.001

The influence of the addition of different concentrations of atRA to the culture medium on chondrogenic differentiation was evaluated via Alcian Blue (AB) staining in 6-day-old cultures treated during the first 2 days of culture (Fig. 1A–C). As shown in Fig. 1B, C, treated cultures presented a reduction in the number of AB-positive chondrogenic aggregates that was associated with expansion of the internodular spaces lacking AB staining (30% of the micromass surface area in controls vs 50% in treated micromasses; Fig. 1D). However, despite this reduction in number, the size of the nodules in the treated cultures was larger and the AB staining was more intense. We observed a 30% increase in the size of the major axis of the AB-positive nodules (n = 100) in the treated cultures evaluated on day 6 of incubation (Fig. 1E). This effect was apparent (Fig. 1B), even at atRA concentrations of 5, 8, and 10 ng/ml, which are below or at the threshold dose that induces cell death (see below), suggesting that these changes were the result of a direct or indirect stimulating effect of atRA on cells that have initiated chondrogenic differentiation rather than secondary to the elimination of progenitors by cell death. Treatments at doses that induce cell death (over 30 ng/ml) caused similar, but more intense effects (Fig. 1C).

Quantification of cell death by PI flow cytometry in 2-day micromasses revealed a potent dose- and time-dependent death-promoting influence of atRA. Addition of atRA at concentrations above 10 ng/ml increased cell death in a concentration-dependent manner. Doses of 30, 50, and 500 ng/ml increased cell death by 292%, 338%, and 400%, respectively (Fig. 1F). In contrast, doses of 5, 8, or 10 ng/ml did not cause a significant increase in cell death. Reducing treatment time to 36 h did not significantly increase cell death employing doses of 10 or 30 ng/ml. Consistent with the apoptotic nature of the dying process, the addition of the pan-caspase inhibitor Q-VD-OPH at 20 µM [6] for 12 h reduced cell death in both controls and atRA-treated cultures (Fig. 1G).

### Cell cycle modifications by atRA

In addition to the proapoptotic effect of atRA described above, the cell cycle was modified in 2-day cultures subjected to distinct atRA concentrations (Fig. 1H). At concentrations of 10, or 30 ng/ml, we observed a significant increase in the percentage of cells in the S-phase, accompanied by minor changes in the percentage of cells in the G0/G1 and G2/M phases. The addition of 50 ng/ml atRA did not affect the cell cycle, except for a moderate increase in the number of cells in the G2/M phase. At an atRA concentration of 200 ng/ml, a significant decrease in the number of S-phase cells accompanied by an increase in the number of G2/M-phase cells were detected.

### Differential expression of genes encoding for the RA synthesizer and catabolizer enzymes *Raldh2* and *Cyp26a1* in the interdigits

**Fig. 2:**
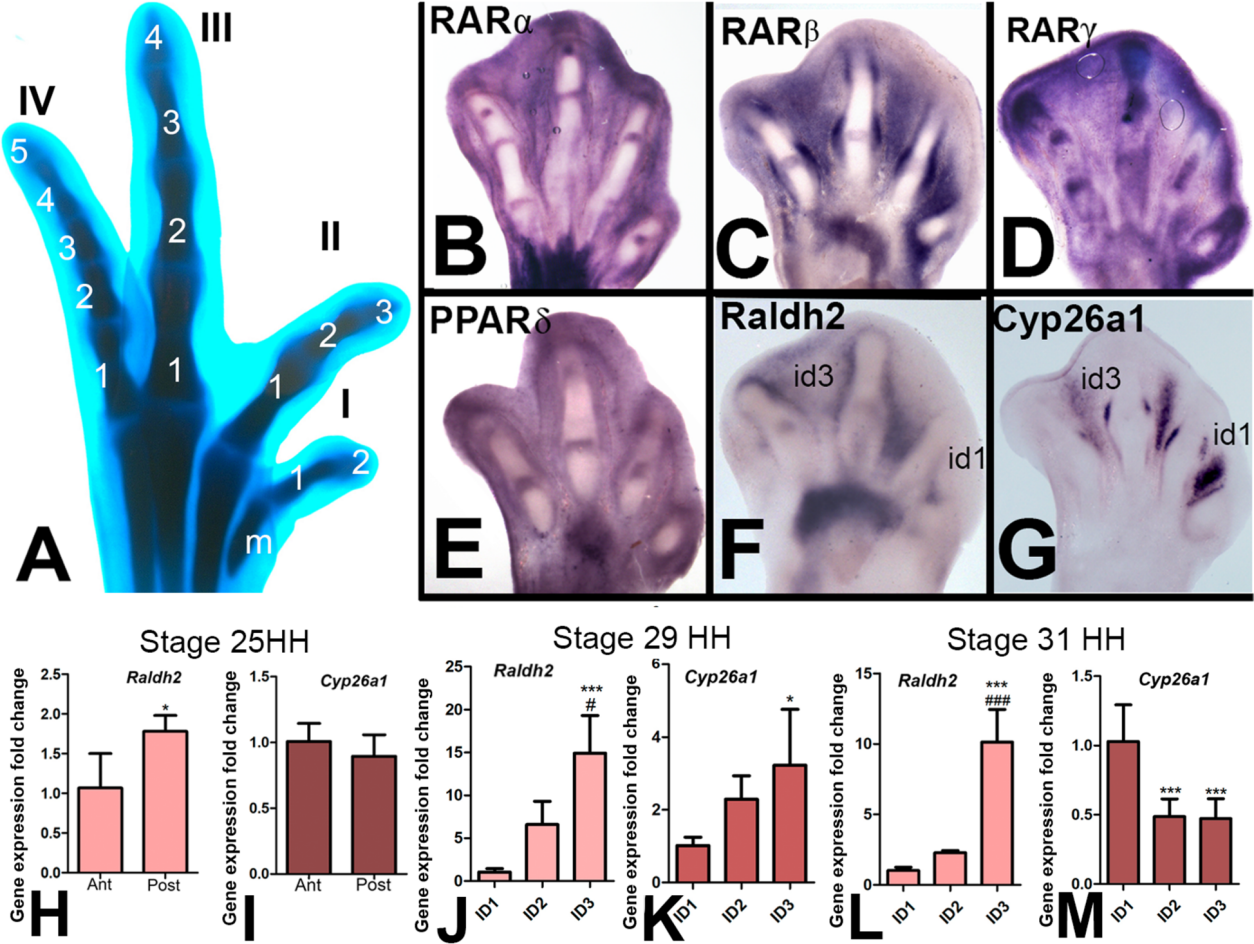
Differential expression of RA signaling pathway genes in the autopod.** A** Embryonic chick leg autopod on day 9 of incubation (stage 35 HH), stained with Alcian Blue, illustrating the normal morphology of the digits. Note the different numbers of phalanxes (2, 3, 4, and 5), and the sizes of the four digits (I, II, III, and IV). Metatarsal (m). **B**–**G** In-situ hybridization images showing the pattern of expression of genes of the RA signaling pathway expressed in the autopod at stages of digit morphogenesis (B, *RARα*; C, *RARβ*; D, *RARγ*; E, *Pparδ*; F, *Raldh2*; and G, *Cyp26a1*). Note the asymmetric expression intensity of *Raldh2* and *Cyp26a* in the first (id1) and third (id3) interdigits. **H**–**M** Scale bar diagrams showing the differential expression levels of *Raldh2* and *Cyp26*a1 in different regions and periods of limb development. *Raldh2* (**H**) and *Cyp26a1* (**I**) relative expression levels in the anterior and posterior halves of the distal mesoderm at stage 25. Student’s *t* test statistical significance: *p < 0.05. *Raldh2* (**J** and **L**), and *Cyp26a1* (K and M) relative expression levels in the first (ID1), second (ID2), and the third interdigits (ID3) at stage 29 HH (J and K) and 31 HH (**L** and **M**). ANOVA statistical significance vs ID1: *p < 0.05; **p < 0.01; ***p < 0.001. ANOVA statistical significance of ID3 vs ID2: ^#^p < 0.05; ^##^p < 0.01; ^###^p < 0.001

The avian autopod has 4 fingers of different sizes and numbers of phalanxes that confer distinctive identities on them (Fig. 2A). Digit 1 is the shortest and has 2 phalanxes; digit 2, has 3 phalanxes; digit 3, has four phalanxes; and, finally, digit 4 has five phalanxes (2–3–4–5).

As shown in Fig. 2B–G, RARs (*α*, *β*, and *γ*) and *PPAR δ* were expressed in the undifferentiated interdigital mesoderm and developing joints, with *RAR β*, showing well-defined domains of high expression in the interdigital mesoderm, developing joints, and the periarticular region of the digits and *RAR γ* being also expressed in the digit rays (Fig. 2C). The RA-synthesizing enzyme *Raldh2* and the RA-degrading enzyme *Cyp26a1* were also highly expressed in the interdigital regions (Fig. 2F, G). Remarkably, these genes showed an apparently differential expression in the three interdigits. Considering previous studies reporting an anteroposterior gradient of retinoids in the first stages of limb formation (stage 18–21; [59]) that was associated with the early patterning of the digits [47], we explored possible differential expression levels of *Raldh2* and *Cyp26a1* at the developmental stages that correspond to the period of digit growth. Comparative qPCR gene expression analysis in anterior, posterior, and interdigital samples of the autopod showed significant regional differences in the expression levels of these genes in the course of digit outgrowth (Fig. 2H–M). By stage 25, digit primordia begin to form, but there is not yet a defined anatomical distinction of digits and interdigits; therefore, the distal 300 µm of the autopod was dissected and the expression of both genes was compared between the anterior and posterior halves. As shown in Fig. 2H, I, while *Cyp26a1* showed a similar expression level in both halves, the expression of *Raldh2* was significantly higher in the posterior halve. By stage 29 digits and interdigits are clearly identifiable, but only the two proximal phalanxes of each digit are formed. Analysis of gene expression revealed a very predominant expression of *Raldh 2* in the third interdigit (almost 20-fold vs the first), and a lower expression in the first interdigit, with interdigit 2, showing an intermediate expression level (Fig. 2J). At this stage *Cyp26a1* showed a much reduced (threefold) but significant increased expression in the third interdigits vs the first (Fig. 2K). However, by stage 31, digit 1 has achieve its final two-phalangeal structure and digits 3 and 4 are still growing to form their distal phalanxes. At this stage *Raldh2* maintained a prominent antero-posterior expression gradient with the first interdigit showing lowest expression and the third interdigit highest expression (Fig. 2L). In an opposite fashion, the RA-degrading enzyme *Cyp26a1* [1], showed highest expression in the first interdigit and lowest in the third (Fig. 2M). No significant differences in the expression of RAR´s and PPAR*δ* genes were observed (not shown). Together, these findings are consistent with the occurrence of an anteroposterior concentration gradient of retinoic acid potentially associated with growth and differentiation of the digit primordia.

### Influence of exogenous RA on digit size

RA promotes or inhibits the growth of limb cartilage in explant cultures in a dose-dependent manner [11, 32]. To determine whether exogenous RA is able to modify the size of the fingers, we performed gain-of-function experiments in which beads incubated with different concentrations of all-trans-retinoic acid were implanted in the interdigital mesoderm. Beads incubated in all-trans-retinoic acid at a low (25–50 µg/ml) or high (100 or 150 µg/ml) concentrations were implanted in the first interdigit which is encompassed by the shortest digits (digits I and II) and the third interdigit which is encompassed by the longest digits (digits III and IV). At stages 24–25, when the rays of digits 1 and 2 are not recognizable, we initially assumed the position of the first interdigits, which was confirmed by observing the embryos 12 h later.

**Fig. 3:**
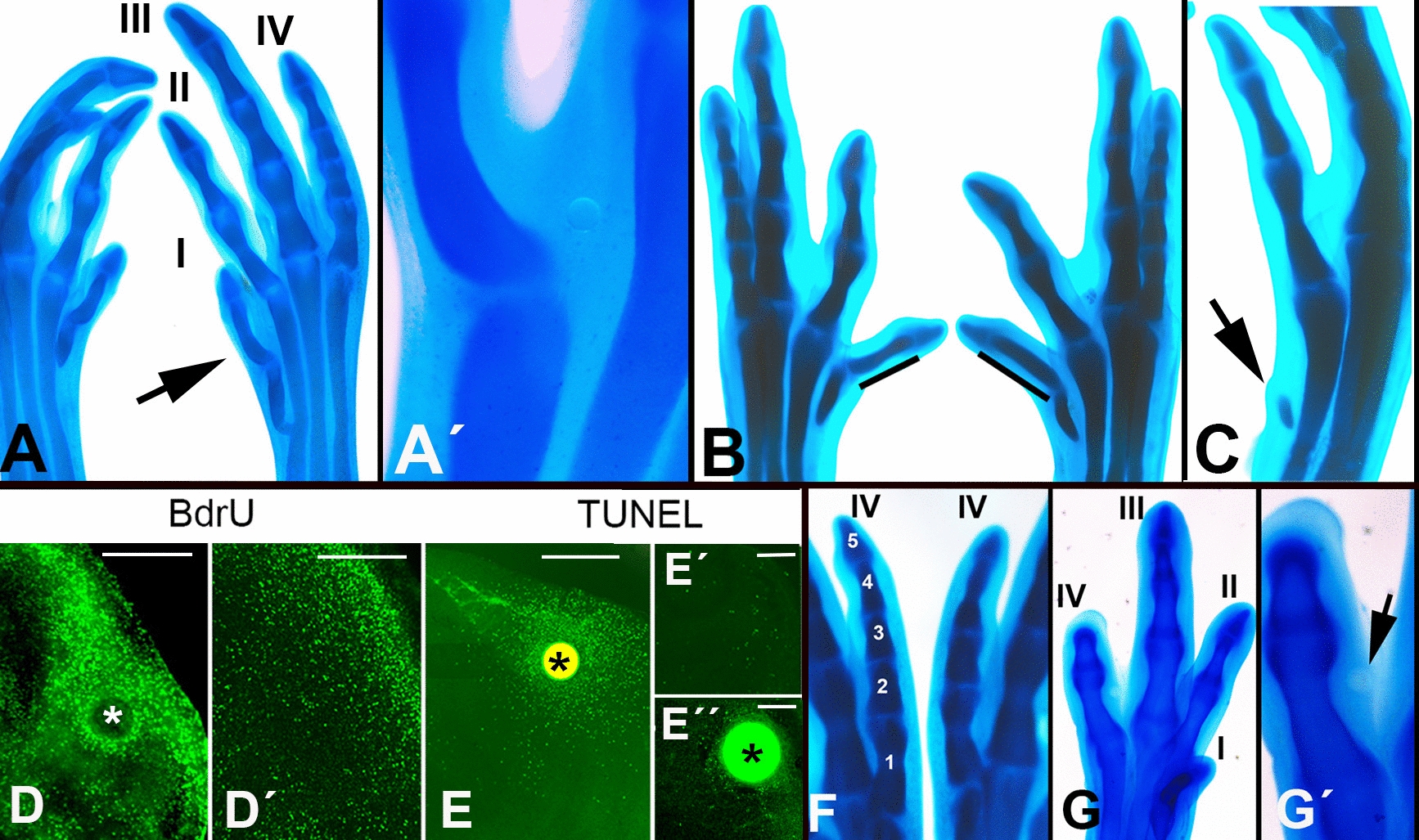
Effects of interdigital application of atRA-beads on the embryonic leg. In all pictures, control and experimental autopods belong to the same embryo. **A** Control (left) and experimental (right) autopods at stage 35 HH after implantation of a low-dose (25 µg/ml) atRA-bead in the first interdigit at stage 25 HH, showing intense enlargement of the first metatarsal and first phalanx (arrow). **A´** Detailed view showing the position of the bead at the end of the experiment. **B** Control (left) and experimental (right) autopods illustrating the enlargement of the first phalanx (indicated by the bar) without changes in the metatarsals. **C** Experimental autopod at stage 35 HH. After implantation of a high-dose (100 µg/ml) atRA-bead in the first interdigit. Note that the autopod has only a rudimentary first metatarsal and no phalanxes (arrow). **D**–**D´** Vibratome sections of experimental (**D**) and control (**D**´) autopods showing differences in BrdU incorporation 8 h after implantation of a low-dose (25 µg/ml) atRA-bead (*) in the first interdigit. **E** TUNEL-positive apoptosis 8 h after implantation of a high-dose (100 µg/ml) atRA-bead (*) in the first interdigit. **E´** shows the absence of TUNEL-positive dead cell at this stage. **E´´** shows the reduced number of TUNEL-positive cells around the bead using low-dose atRA (50 µg/ml). **F** Control (left) and experimental (right) Digit IV stained with Alcian Blue after implantation of an atRA-bead (100 µg/ml) in the third interdigit. Note that digit phalanxes 3 and 4 are substituted by an undivided cartilaginous piece in the experimental leg (observed in 6 out of 20 treated embryos). **G** Severe truncation of digit IV after interdigital implantation of a bead (*) incubated with 200 µg/ml atRA (observed in all experimental limbs). **G´** Magnification view of (G) showing the position of the bead (arrow). Scale bars in (D, D´, and E) = 200 µm. Scale bars in (E´, E´´) = 80 µm

Low-atRA-concentration beads implanted in the first interdigits at stages 24/25, but not at later stages, modified the size of digit 1. This effect was detected in only a small number of experiments (n = 16 out of 92 treated embryos) but was never observed after implantation of control beads (n = 60), suggesting that the variability is due to small, unavoidable differences in the localization of the RA-beads. By in-situ hybridization of different retinoic acid target genes, we observed that the transcriptional influence of RA-beads implanted in the limb at the studied stages, extends 100–200 µm around the bead. As shown in Fig. 3A, digit 1 was longer in the treated embryos than in the contralateral control limb. In most cases (n = 11), these digits showed elongation of the first metatarsal, which may appear fused with the second metatarsal (Fig. 3A). This alteration was often accompanied by elongation of the first phalanx. In a reduced number of cases (n = 5), elongation was appreciable only in the first phalanx (Fig. 3B). Measurement of the size of the skeletal parts of the experimental toes on day 8.5 (35HH) of incubation showed that overgrowth of the first toe was most affected at the level of the first metatarsal (overgrowth ranged from 20 to 100% compared to the contralateral feet, with a mean of 46%). Overgrowth of the first phalanx was more moderate (overgrowth ranged from 16 to 52%, with a mean of 26%). No significant size changes were observed in the distal phalanx. Digit 2 was unaffected in these experiments except in a few cases (n = 2). High-concentration atRA-beads (over 100 μg/ml), inhibited digit growth, which appeared truncated in all treated embryos (n = 20; Fig. 3C). Analysis of cell proliferation and cell death via BdrU incorporation and TUNEL labeling, revealed that low-atRA-concentration beads promoted the proliferation of interdigital progenitors (Fig. 3D-–D´), whereas high-concentration beads promoted apoptosis (Fig. 3E–E´´).

Implantation of low-atRA-concentration beads in the third interdigit failed to increase the size of adjacent digits 3 and 4, which in birds are physiologically much longer than digits 1 and 2, suggesting that growth signaling in this space is physiologically saturated. In turn, as observed in the first interdigit, high-concentration atRA-beads (100 or 150 µg/ml) induced moderate truncations of digit IV consisting of loss, or reduction in size, of a single phalanx, accompanied by the loss of an interphalangeal joint (6 of 20 treated embryos; Fig. 3F). Higher concentrations (200 µg/ml) caused truncations, characterized by the loss of two or three phalanges (Fig. 3G).

### Inhibition of RA synthesis decreased the size and number of phalanxes in the fingers

The above-described observations suggest the influence of local RA concentrations on determining the size of the fingers. To test this hypothesis, we designed experiments in which RA synthesis was locally inhibited via treatment with Citral, a potent inhibitor of RA synthesis that acts via the inhibition of Raldh2 [63]. Local treatments were performed via implantation of SM2 beads (BioRad) incubated for 1 h. in 200 µM Citral. We chose the third interdigit for the treatments, because it has the largest size and highest *Raldh2* expression level.

**Fig. 4:**
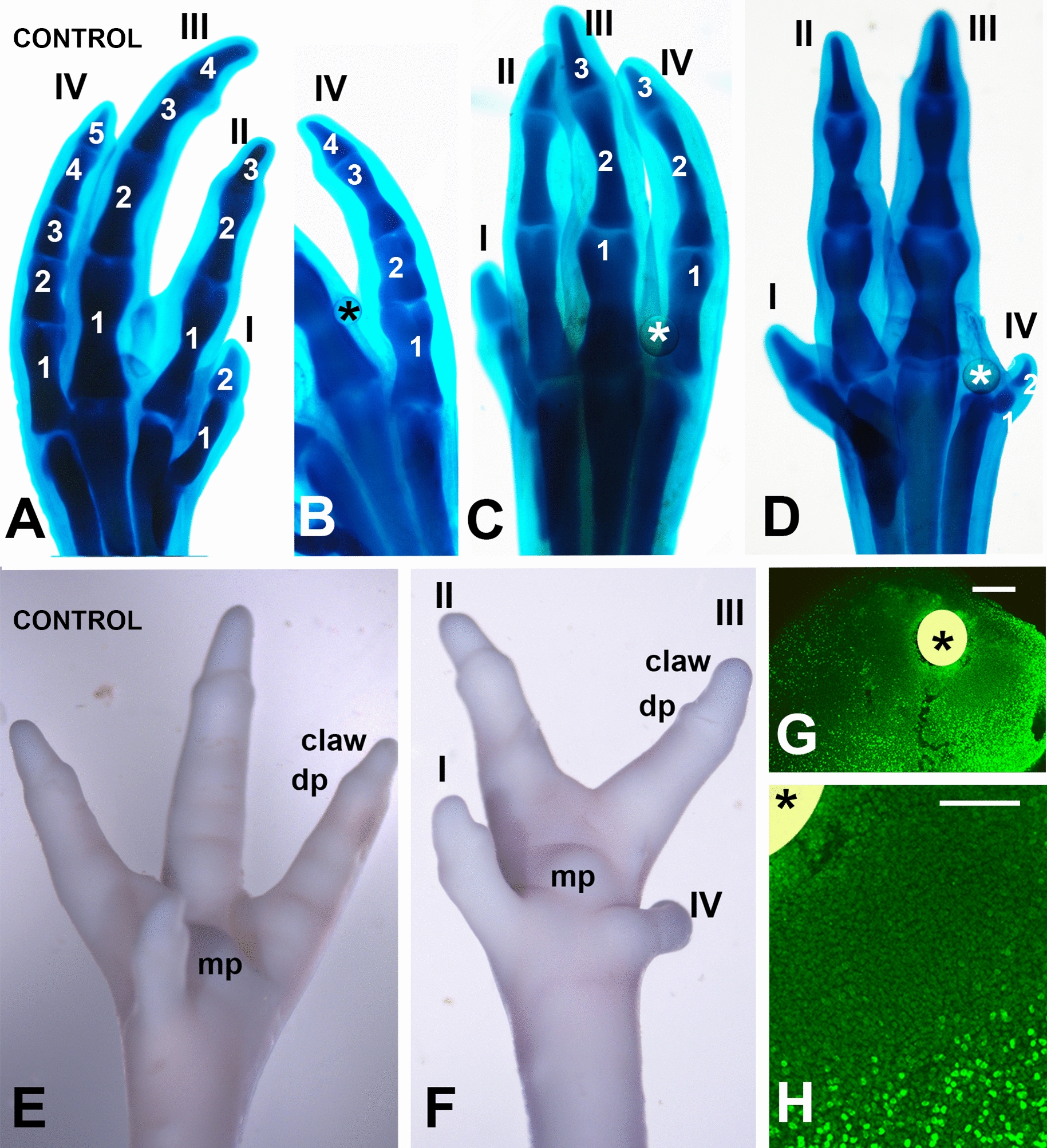
Effects of interdigital application of beads (*) incubated in 200 µM Citral, at stage 27 HH. **A** Control autopod at stage 35 HH, displaying the characteristic phalangeal formula 2–3-4–5 in digits I, II, III, and IV. **B**–**D** Illustrate three levels of phalangeal loss following interdigital implantation of Citral beads (*). Note the autopods with phalangeal formulas 2–3-4–4 (**B**; observed in 6 out of 29 autopods with phenotype); 2–3–3–3 (**C**; observed in 6 out of 29 autopods with phenotype) and 2–3–3–2 (**D**; observed in 8 out of 29 autopods with phenotype). **E**, **F** Surface view of control (**E**) and experimental (**F**) autopods (stage 35 HH) following interdigital implantation of a Citral-bead (F) to illustrate the maintenance of normal morphology of the distal phalanxes, including the claw, digit pads (dp), and metatarsal pad (mp), despite of the reduced size of digits III and IV. **G**, **H** Low magnification (**G**) and detailed view (**H**) of a vibratome section of the experimental autopod showing BrdU incorporation 8 h after implantation of a Citral-bead (*) in the third interdigit. Scale bars, G = 100 µm; H = 50 µm

Consistent with the antichondrogenic influence of Citral on limb explants [60], local application of Citral beads resulted in a reduction in the size and number of phalanges of digits 3 and/or 4, in 41 of 67 experimental embryos (Fig. 4A–D). The reduction in digit size was related to the number of missing phalanges that was more prominent in digit 4 than in digit 3. Thus, a digit 3 with only 3 phalanges, which is the normal pattern for digit 2, was observed in a total of 25 embryos (out of 67; Fig. 4C). In the remaining embryos, digit 3 was normal except in two cases, where there were only 2 phalanges (not shown). Concerning digit 4, 6 autopods had 4 phalanges instead of 5 (similar to the morphology of digit 3; Fig. 4B); 6 autopods had a 3-phalangeal digit 4 (similar to normal digit 2; Fig. 4C); 8 autopods had a 2-phalangeal digit 4 (similar to normal digit 1; Fig. 4D); 5 autopods had a digit 4 with only one phalanx; and, in four cases phalanges were absent. Importantly, the morphology of the digits with reduced number of phalanxes was in most cases normal, including the appearance of a normal claw at the distal tip (Fig. 4E, F), suggesting that these changes were associated with the dysregulation of physiological growth signals rather than unspecific truncations secondary to the disruption of cartilage differentiation or abnormal degeneration.

At the tissue level, alterations induced by Citral beads consisted of intense inhibition of cell proliferation (Fig. 4G, H), whereas apoptosis was reduced but not abolished.

## Discussion

RA forms a complex signaling cascade of major relevance in limb morphogenesis and regeneration [9, 16, 31, 37, 68, 72, 73]. Its function extends from the induction of the limb bud primordium at the flank of the embryonic body to the differentiation of the skeletal tissues in the postnatal period [57]. Furthermore, its local concentration is critical for normal development [72], and both, excess and deficiency of RA cause multiple malformations [58]. The study of retinoic acid has received considerable attention from developmental biologists, but many aspects of its functionality are not yet fully understood.

Our in vitro study illustrates the multifunctional properties of retinoic acid signaling reported in the embryonic limb and other developing models [32, 51, 62, 66]. We show that atRA added to the cultures has dual contradictory effects, promoting both proliferation and apoptosis in the undifferentiated progenitors but simultaneously stimulating the growth and maturation of differentiating cartilage nodules. The inhibitory effect of the pan-caspase inhibitor Q-VD-OPH [6] confirms the apoptotic nature of cell death induced by atRA treatment.

The differential effects of atRA are likely due to the activation of different receptors. Canonical AR signaling is mediated by the RAR receptors α, β, and γ, but RA also binds to the orphan nuclear receptor PPARβ/δ. The latter has been linked to cell survival, while proapoptotic function has been associated with signaling via RARs [54]. This interpretation is in agreement with the syndactylous phenotypes observed in compound mutants of RAR receptors [21, 39]. According to the Schug study [54], two cytoplasmic AR-binding proteins, CRABP-II and FABP5, selectively regulate AR binding to RAR or PPARβ/δ, thereby directing the proapoptotic vs proliferative functions of AR. In this study, we observed a relatively similar RARs and PPARβ/δ gene expression patterns in the developing autopod, including the interdigits and joints. Furthermore, our unpublished qPCR study confirmed the expression of both CRABP-II and FABP5 in limb mesoderm. However, our observations do not provide evidence for differential expression domains of the receptors according to potential differential functions. Future functional studies specifically silencing the different receptors and/or cytoplasmic RA-binding proteins are required to clarify this question.

The role of RA repressing the differentiation of skeletal tissues is widely supported by previous studies and reinforced by the present results. However, our observations are also consistent with a role of RA in promoting cartilage differentiation. These findings are in agreement with those of Dranse et al. [13], who reported that, under the influence of RA skeletal progenitors are maintained in an undifferentiated state waiting for the appropriate signals to initiate differentiation. This feature may explain why both excess and defects in the RA generate skeletal malformations. The excess of RA maintained over a prolonged time period abrogates differentiation resulting in excessive cell death, but the absence of RA prevents the formation of the appropriate number of progenitors for subsequent differentiation, resulting in equally defective skeletal development. However, the outcome of the micromass cultures subjected to RA treatments is also indicative of a positive effect of RA treatments on cartilage differentiation. As suggested in other experimental settings [38], this may be mediated via the upregulation of BMP genes as they promote both cartilage growth and differentiation and interdigital apoptosis during digit morphogenesis [35].

The role of RA as signaling molecule that direct growth and skeletogenesis has been demonstrated for the early limb primordia [41, 76], yet whether this role is permissive or instructive has not been determined (see [33]). In addition, experiments at the end of the last century, demonstrated that exogenous RA is able to mimic the function the ZPA in the regulation of the antero-posterior axis of the limb, although it was no clear if this is a physiological function of RA [47]. Our present study suggests an additional role for RA in the establishment of the asymmetric size of the avian digits that has not been detected in mouse embryos deficient in Raldh2 rescued by transient administration of RA to pregnant mice [76].

According to our findings, the dual effect of atRA promoting proliferation and cell death may contribute to establishing the digit length and structure (i.e., the “identity”) and the digit/interdigit pattern in the autopode. The digit pattern of tetrapods is regulated by a locally patterned distribution of signaling molecules in the autopod [3, 28, 56], and shows major differences among vertebrates conferring sophisticated specializations to different species (e.g., swim, fly, grasp, or run, etc.). In relation to evolutionary changes in the pentadactyl limb archetype, the chick has lost one toe in the foot and the remaining four have different sizes and numbers of phalanges (Fig. 2A), providing an optimal model to evaluate signals that regulate finger growth [56]. The differential expression of Raldh2/Cyp26 in the three interdigits of the chick leg autopod, observed here, together with the structural (number of phalanxes) and size modifications of the digits in gain and loss of function experiments induced by local application of atRA or Citral, respectively, suggest a role of RA signaling in the establishment of the digit “morphology/identity”. This interpretation is in full agreement with the proposed role of interdigits as signaling centers that determine the identity of the digits at advanced stages of development [10] and with the reported role of RA in the regulation of Barx1, which in turn direct the position of the joints [70].

Currently, classical studies on limb development have revealed that the anteroposterior axis of the limb bud is patterned via the activation of Sonic Hedgehog signaling by the ZPA, a mesodermal region located in the posterior margin of the early limb bud (see, [27]). However, this initial instruction must be locally executed by signals that regulated the growth and differentiation of progenitors. The players proposed for this function include, Activin/BMP [45, 61], Wnt/FGF [53, 71], and Hoxd genes and Gli3 [28]. Our findings identified RA as a complementary signal in the molecular network responsible for establishing digit shape and structure. In this regard, it is worth emphasizing, the important transcriptional interactions between RA signaling and the expression of various miRNAs [19, 26] that in turn might regulate factors of major importance during in limb morphogenesis and tissue differentiation (including the remodeling of the interdigital tissue [20]. Findings obtained from genetic analysis of the synpolydactyly mouse mutant [31] suggest that the gradient distribution of Raldh2 in the embryonic chick autopod may be associated with the activity of HoxD13, a homeobox gene with a predominant expression in the posterior region of the autopod that occupies the most 5′ position of the Hox D cluster.

A final consideration of this study is the resemblance of some of our experimental autopods with specialized digit patterns present in other tetrapods (see [65]). On the basis of our findings, it is tempting to speculate about an evolutionary contribution of RA signaling to digit morphogenesis. Previous studies have shown numerous transcriptional differences associated with distinct autopodial morphologies in tetrapods [64]. In our experiments, the presence of a three-phalangeal digit 3 and two-phalangeal digit 4 (2–3–3–2 phalangeal pattern) observed in a number of Citral treated embryos (Fig. 4) was reminiscent of the normal autopod morphology in pig embryos [55, 64].

## Supplementary Information

Additional file 1.

## Data Availability

The data sets analyzed during the current study are available in the Figshare repository, with the identifier 10.6084/m9.figshare.28507340.
