# Supplementary material for: Retinoic acid regulates the proliferation, differentiation, and cell death of limb skeletal progenitors, contributing to establish the size and identity of the digits
Source: EvoDevo. 2025 Jul 9;16:11. doi: 10.1186/s13227-025-00248-4 (PMC12239497; doi:10.1186/s13227-025-00248-4)
Supplement: Supplementary file 1 — Additional file 1. [file 13227_2025_248_MOESM1_ESM.docx]

| **qPCR Chicken specific primers.** | | | |
| --- | --- | --- | --- |
| ***Gene*** | **NCBI Ref. Seq.** | **Fwd. Primer** | **Rev. Primer** |
| *Ch. Cyp26a1* | NM_001001129 | cctctccaaccttcacaacg | acctcctcttggatgacagg |
| *Ch. Gapdh* | NM_204305 | ggtggccatcaatgatcc | gttctcagccttgacagtgc |
| *Ch. PPARD* | NM_204728 | ggcgttgtagatgtgcttgg | ggaggcagagaagaggaagc |
| *Ch. RPL13* | NM_204999 | aactcaagatggcaactcagc | aaggccttgaagttcttctcc |
| *Ch. RALDH2* | NM_001397808 | tgaggtattacgctggttgg | tgtccacacactccgatgg |
| *Ch. RARA* | NM_204536 | gctcacagacctggtgttcg | gcagtttgtccaccttgtcg |
| *Ch. RARB* | NM_205326 | cgaactcagatgcacaatgc | tccacagatcaagcagatgg |
| *Ch. RARG* | XM_046905006 | ctgggcaaatacaccatgagc | gcatttggtggccaactcg |

Supplementary Table 1: qPCR Chicken specific primers.
